# P03-001 - PFAPA and MEFV genes

**DOI:** 10.1186/1546-0096-11-S1-A196

**Published:** 2013-11-08

**Authors:** F Salehzadeh, M Vahedi, S Jahangiri, S Hosseiniasl

**Affiliations:** 1 Pediatric Rheumatology, Bouali Hospital, Iran, Islamic Republic Of; 2 Pediatric Rheumatology, ARUMS, Iran, Islamic Republic Of; 3 Genetic, Emam Khomeini Hospital, Ardabil, Iran, Islamic Republic Of

## Introduction

Marshall Syndrome or PFAPA is an inflammatory periodic disease characterized by periodic fever, aphthous stomatitis, pharyngitis and cervical adenitis. Restless, headache, abdominal pain, vomiting, hepatosplenomegaly and arthralgia are less common symptoms seeing in this disease. The diagnosis is established on the basis of clinical criteria that require the presence of a recurrent fever of early onset (<5 years) and ≥1 of the 3 associated symptoms (aphthosis, cervical adenitis, and pharyngitis), in the absence of upper respiratory tract infections and cyclic neutropenia.

## Objectives

Although PFAPA is an auto inflammatory disease, it doesn't have genetic basis such as other periodic fevers. This study evaluates the 12 common MEFV gene mutations in patients with PFAPA syndrome.This study evaluates the 12 common MEFV gene mutations in patients with PFAPA syndrome.

## Methods

21 patients with PFAPA syndrome who had diagnostic criteria were enrolled in this study and 12 common MEFV gene mutations were evaluated in them. The 12 most common MEFV gene mutations (P369S, F479L, M680I (G / C), M680I (G / A), I692del, M694V, M680I, K695R, V726A, A744S, R761H, E148Q) were analyzed by using amplification refractory mutation system for 11 of the first and the PCR was performed for E148Q.

## Results

The age of patients was between 6 months to 14 years old, and 15 were male. Seven patients had heterozygote and one had compound heterozygote (K695R, V725A) mutation. There were 4 alleles M694V, 3 alleles V726A, 1 allele E148Q and 1 allele K694R. No significant difference between mutated patients with non-mutated in symptoms like aphthous and stomatitis, duration of attacks, episodes of fever and response to treatment. Gaslini score test was not helpful to predict the probability of gene mutations.

## Conclusion

About 30 percent of patients had MEFV gene mutations but these mutations don't play a main role in presentation of PFAPA symptoms.

## Competing interests

None declared.

